# Age, Sex and Previous Comorbidities as Risk Factors Not Associated with SARS-CoV-2 Infection for Long COVID-19: A Systematic Review and Meta-Analysis

**DOI:** 10.3390/jcm11247314

**Published:** 2022-12-09

**Authors:** Kin Israel Notarte, Maria Helena Santos de Oliveira, Princess Juneire Peligro, Jacqueline Veronica Velasco, Imee Macaranas, Abbygail Therese Ver, Flos Carmeli Pangilinan, Adriel Pastrana, Nathaniel Goldrich, David Kavteladze, Ma. Margarita Leticia Gellaco, Jin Liu, Giuseppe Lippi, Brandon Michael Henry, César Fernández-de-las-Peñas

**Affiliations:** 1 Department of Pathology, Johns Hopkins University School of Medicine, Baltimore, MD 21205, USA; 2 Department of Biostatistics, State University of Maringa, Maringá 87020-900, Brazil; 3 Faculty of Medicine and Surgery, University of Santo Tomas, Manila 1008, Philippines; 4 New York Medical College, Valhalla, NY 10595, USA; 5 Department of Biomedical Engineering, Johns Hopkins University, Baltimore, MD 21218, USA; 6 Section of Clinical Biochemistry, University of Verona, 37129 Verona, Italy; 7 Clinical Laboratory, Division of Nephrology and Hypertension, Cincinnati Children’s Hospital Medical Center, Cincinnati, OH 45229, USA; 8 Department of Physical Therapy, Occupational Therapy, Physical Medicine and Rehabilitation, Universidad Rey Juan Carlos, 28933 Madrid, Spain

**Keywords:** post-COVID-19 condition, long COVID-19 symptoms, risk factors, sex, age, co-morbidity

## Abstract

Identification of predictors of long COVID-19 is essential for managing healthcare plans of patients. This systematic literature review and meta-analysis aimed to identify risk factors not associated with Severe Acute Respiratory Syndrome Coronavirus 2 (SARS-CoV-2) infection, but rather potentially predictive of the development of long COVID-19. MEDLINE, CINAHL, PubMed, EMBASE, and Web of Science databases, as well as medRxiv and bioRxiv preprint servers were screened through 15 September 2022. Peer-reviewed studies or preprints evaluating potential pre-SARS-CoV-2 infection risk factors for the development of long-lasting symptoms were included. The methodological quality was assessed using the Quality in Prognosis Studies (QUIPSs) tool. Random-effects meta-analyses with calculation of odds ratio (OR) were performed in those risk factors where a homogenous long COVID-19 definition was used. From 1978 studies identified, 37 peer-reviewed studies and one preprint were included. Eighteen articles evaluated age, sixteen articles evaluated sex, and twelve evaluated medical comorbidities as risk factors of long COVID-19. Overall, single studies reported that old age seems to be associated with long COVID-19 symptoms (n = 18); however, the meta-analysis did not reveal an association between old age and long COVID-19 (n = 3; OR 0.86, 95% CI 0.73 to 1.03, *p* = 0.17). Similarly, single studies revealed that female sex was associated with long COVID-19 symptoms (n = 16); which was confirmed in the meta-analysis (n = 7; OR 1.48, 95% CI 1.17 to 1.86, *p* = 0.01). Finally, medical comorbidities such as pulmonary disease (n = 4), diabetes (n = 1), obesity (n = 6), and organ transplantation (n = 1) were also identified as potential risk factors for long COVID-19. The risk of bias of most studies (71%, n = 27/38) was moderate or high. In conclusion, pooled evidence did not support an association between advancing age and long COVID-19 but supported that female sex is a risk factor for long COVID-19. Long COVID-19 was also associated with some previous medical comorbidities.

## 1. Introduction

Long COVID-19 is a term used for defining the persistence of signs and symptoms in people who recovered from an acute Severe Acute Respiratory Syndrome Coronavirus 2 (SARS-CoV-2) infection [1]. Long COVID-19 is defined by the World Health Organization (WHO) as: “post-COVID-19 condition, occurs in individuals with a history of probable or confirmed SARS-CoV-2 infection, usually 3 months from the onset, with symptoms that last for at least 2 months and cannot be explained by an alternative diagnosis [2].” Several meta-analyses investigating the prevalence of post-COVID-19 symptoms have been published, concluding that around 30–50% of subjects who recover from a SARS-CoV-2 infection develop persistent symptoms lasting up to one year [3,4]. A recent meta-analysis concluded that two years after the initial spread of coronavirus disease 2019 (COVID-19), up to 42% of infected patients experienced long COVID-19 symptoms [5].

Different narrative reviews have mentioned prognostic aspects, but no comprehensive synthesis has been provided so far [6,7,8,9]. Identification of potential risk factors associated with post-COVID-19 syndrome is important since identifying individuals at higher risk can guide management healthcare plans for these patients and reorganize healthcare accordingly. Iqbal et al. tried to pool data, but these authors were only able to pool prevalence data of post-COVID-19 symptomatology, but not risk factors [10]. All these narrative reviews have suggested that female sex, old age, higher number of comorbidities, higher viral load, and greater number of COVID-19 onset symptoms can be potential risk factors for long COVID-19 [6,7,8,9,10]. However, no systematic search or assessment of methodological quality was conducted in these reviews [6,7,8,9,10]. Two meta-analyses have recently focused on risk factors of long COVID-19. Maglietta et al. identified that female sex was a risk factor for long COVID-19 symptoms, whereas a more severe condition at the acute phase was associated just with long COVID-19 respiratory symptoms [11]. Thompson et al. found that old age, female sex, white ethnicity, poor pre-pandemic health, obesity, and asthma can predict long COVID-19 symptoms [12]. However, this analysis included just studies from the United Kingdom, and used the definition of long COVID-19 proposed by the National Institute for Health Care and Excellence (NICE) [13].

Accordingly, current evidence on risk factors associated with post-COVID-19 condition is heterogeneous. Risk factors can be classified as pre-infection (e.g., age, sex, previous comorbidities, and previous health status) and infection-associated (e.g., disease severity, symptoms at onset, viral load, hospitalization stay, and intensive care unit admission) factors. The current systematic review and meta-analysis aimed to identify factors not directly associated with acute SARS-CoV-2 infection (i.e., pre-infection factors) such as age, sex, and previous medical comorbidities, which may predict the development of long COVID-19 symptomatology.

## 2. Methods

This systematic literature review and meta-analysis aiming to identify the association of age, sex, and comorbidities as predictive factors for development of long COVID-19 was conducted following the Preferred Reporting Items for Systematic reviews and Meta-Analyses (PRISMA) statement of 2020 [14]. We also followed specific criteria recommended by Riley et al. to systematic reviews and the meta-analysis of prognostic factor studies [15]. The review study was prospectively registered in the Open Science Framework (OSF) database (https://osf.io/79pdg).

### 2.1. Search Strategy and Selection Criteria

Two different authors performed an electronic search for articles published up to 15 September 2022 MEDLINE, CINAHL, PubMed, EMBASE, and Web of Science databases, as well as on preprint servers medRxiv and bioRxiv, using the following search terms: “long COVID-19” OR “post-acute COVID” OR “post-COVID-19 condition” OR “long hauler” AND “age” OR “sex” OR “medical comorbidities” OR “transplant” OR “obesity” OR “diabetes” OR “hypertension” OR “pulmonary disease” OR “asthma” OR “chronic obstructive pulmonary disease”. The search was focused on the medical comorbidities likely associated with a more severe COVID-19 condition. Combinations of these search terms using Boolean operators are outlined in Table 1.

The Population, Intervention, Comparison. and Outcome (PICO) principle was used to describe the inclusion and exclusion criteria: 

Population: Adults (>18 years) infected by SARS-CoV-2 and diagnosed with real-time reverse transcription-polymerase chain reaction (RT-PCR) assay. Subjects could have been hospitalized or not by SARS-CoV-2 acute infection.

Intervention: Not applicable.

Comparison: People infected by SARS-CoV-2 who did not develop long COVID-19 symptoms.

Outcome: Collection of long COVID-19 symptoms developed after an acute SARS-CoV-2 infection by personal, telephone, or electronic interview. We defined post-COVID-19 condition according to Soriano et al. [2], where “post-COVID-19 condition occurs in individuals with positive history of probable or confirmed SARS-CoV-2 infection, usually 3 months from onset of COVID-19, with symptoms that last for at least 2 months and cannot be explained by alternative diagnosis.” We considered any long COVID-19 symptom appearing after the infection, e.g., fatigue, dyspnea, pain, brain fog, memory loss, skin rashes, palpitations, cough, and sleep problems. Results should be reported as odds ratio (OR), hazards ratio (HR), or mean incidence of the symptoms.

### 2.2. Screening Process, Study Selection, and Data Extraction

This review included observational cohort, cross-sectional, and case-control studies whether presence of risk factors for development of symptoms appearing after an acute SARS-CoV-2 infection were investigated in COVID-19 survivors, either hospitalized or non-hospitalized. The current review was limited to human studies and English language papers. Editorials, opinion, and correspondence articles were excluded.

Two authors screened title and abstract of publications obtained from database search and removed duplicates. Full text of eligible articles was retrieved and analyzed. The following data were extracted from each study: authors, country, design, sample size, age range, assessment of symptoms, long COVID-19 symptoms, and effect (measure) of risk factor studied. Discrepancies between reviewers in any part of the screening and data extraction process were resolved by a third author.

### 2.3. Risk of Bias

The Quality in Prognosis Studies (QUIPSs) tool was used to determine the risk of bias (RoB) of the studies [16]. The QUIPS consists of six domains such as study participation, study attrition, prognostic factor measurement, outcome measurement, adjustment for other prognostic factors, and statistical analysis. RoB was initially evaluated by two authors. If there is disagreement, a third researcher arbitrated a consensus decision. Risk of bias was scored as low, moderate, or high as follows: 1 if all domains are classified as having low RoB, or just one as moderate RoB, the paper was classified as low RoB (green); 2 if one or more domains are classified as having high RoB; or ≥3 if all domains have moderate RoB, the paper was classified as high RoB (red). All papers in between were classified as having moderate RoB (yellow) [17].

### 2.4. Data Synthesis

We conducted a qualitative synthesis of data for those risk factors where the heterogeneity of results did not permit to perform a meta-analysis. We only included articles in the meta-analysis that followed the Soriano et al. definition of post-COVID-19 condition [2], hence meta-analysis was possible for age and sex. 

To synthesize the association between age and sex with post-COVID-19 condition, random-effects meta-analyses were performed using MetaXL software ( https://www.epigear.com/index_files/metaxl.html) to estimate weighted mean differences (for age) and pooled odds ratios (ORs) with 95% confidence intervals (CIs) for sex and age above 60 years (old adults). A *p*-value < 0.05 was considered statistically significant. Given the heterogeneity expected, a random-effects model was employed. Measures of heterogeneity such as the I square statistics and Cochran’s Q test statistic and *p*-value are also reported. When each age group was reported using median and interquartile range values, the method described by Wan was used for transformation in mean and standard deviation.

## 3. Results

### 3.1. Study Selection 

**Figure 1:**
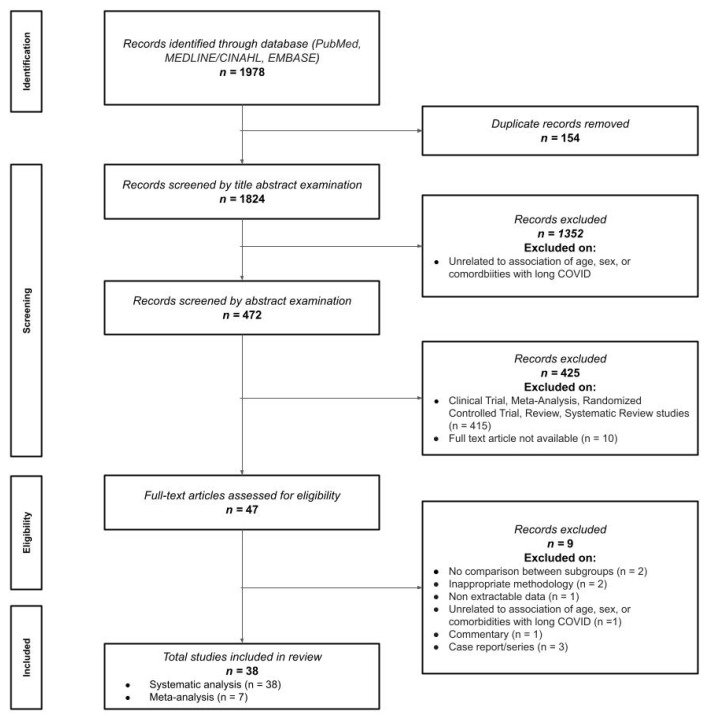
Preferred Reporting Items for Systematic reviews and Meta-Analyses (PRISMA) flow diagram.

The electronic search allowed to initially identify 1978 titles for screening. After removing duplicates (n = 154) and papers not directly related to risk factors (n = 1352), 472 studies remained for abstract examination. Four hundred and twenty-five (n = 425) were excluded after reading the abstract, thus leading to a total of 47 text articles for eligibility (Figure 1). Nine articles were excluded because there were no comparisons between subgroups (n = 2) [18,19], inappropriate methodology (n = 2) [12,20], data not extractable (n = 1) [21], unrelated to association of risk factors (n = 1) [22], and type of literature commentary, case reports, and case series (n = 3) [23,24,25]. A total number of 37 peer-reviewed studies and one pre-print study were finally included [26,27,28,29,30,31,32,33,34,35,36,37,38,39,40,41,42,43,44,45,46,47,48,49,50,51,52,53,54,55,56,57,58,59,60,61,62,63]. All papers could be included in qualitative analysis, whereas seven of these could also be pooled in the meta-analysis. 

### 3.2. Age and Post-COVID-19 Condition

A total of 18 articles, including 819,884 COVID-19 survivors analyzed age as a risk factor for developing long COVID-19 symptoms (Table 2) [26,27,28,29,30,31,32,33,34,35,36,37,38,39,40,41,42,43]. Four articles used percentages [27,33,34,38], five used means [26,31,40,41,43], seven OR [28,29,30,32,36,37,39], one adjusted OR (aOR) [35], and one adjusted hazard ratio (aHR) [42]. Eight articles included population samples aged ≥50 years old [27,28,32,35,37,38,40,41], eight individuals aged between 40 and 49 years [26,29,31,33,34,39,42,43], and one a population between 18 and 64 years [27]. Two studies included children aged 10–12 years [30,36], but data from these age groups were not considered in the main analyses.

Overall, most articles observed that old age was associated with long COVID-19 symptoms [26,28,29,31,33,34,35,37,38,39,40,41,43]. Contrastingly, Peghin et al. did not find an association between age and long COVID-19 symptoms [32]. Subramanian et al. stated that adults aged >70 years displayed lower risk of developing long COVID-19 symptoms than those aged 30–39 years [42]. 

**Figure 2:**
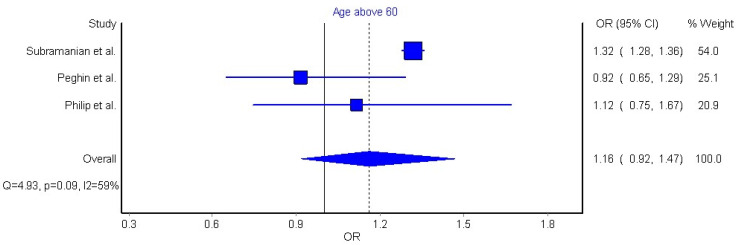
Pooled analysis of odds ratio (OR) for the association between age older than 60 years and the presence of long COVID-19 symptoms [32,42,44].

**Figure 3:**
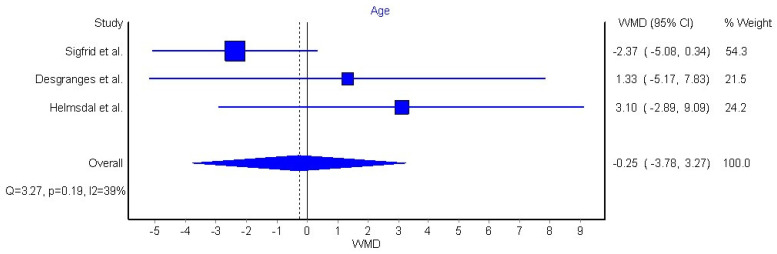
Pooled weighted mean difference (WMD) for the association between age as continuous variable and the presence of long COVID-19 symptoms [43,45,46].

Three articles (n = 30,371 patients) were included in the meta-analysis based on their similar study design, study outcomes, and long COVID-19 definition [32,42,44]. We grouped individuals aged over 60 years old, since this age group is considered to be at higher risk of severe COVID-19. The meta-analysis did not reveal a significant association between old age and long COVID-19 symptomatology (OR 0.86, 95% CI 0.73 to 1.03, Q = 3.54, *p* = 0.17, I2: 44%, Figure 2). Another three articles reporting data as mean (with their standard deviation) or median (interquartile range) were also pooled [42,45,46]. We pooled these data through a random effects model, resulting in a non-significant weighted mean difference (WMD) of −0.25 (95% CI −3.78 to 3.27, Q = 3.27, *p* = 0.19, I2: 39%, Figure 3).

### 3.3. Sex and Post-COVID-19 Condition

A total of 16 articles [32,42,43,44,45,46,47,48,49,50,51,52,53,54,55,56] including 504,044 COVID-19 survivors were used in the analysis of sex as a risk factor for developing long COVID-19 symptomatology (Table 3). Data were presented as OR, aOR, HR, aHR, and percentage. Seven articles used OR [32,47,48,49,50,51,52], two used aOR [45,53], another two used both OR and aOR [46,54], while three articles used percentage [43,44,55], one article used both percentage and OR [56], and one article used both HR and aHR [42].

Fourteen articles observed that female sex (n = 276,953) was associated with higher risk of long COVID-19 [32,42,45,46,47,48,49,50,51,52,53,54,55,56], whilst two articles (n = 475) reported that female sex was not associated with higher risk of long COVID-19 [43,44]. 

**Figure 4:**
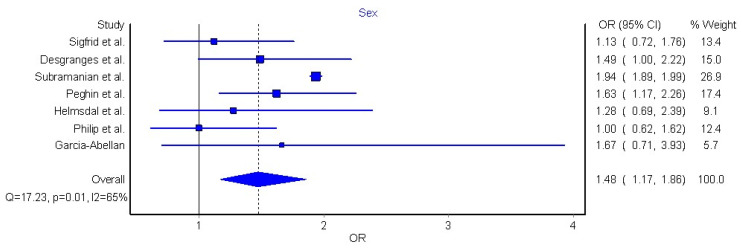
Pooled analysis of odds ratio (OR) for the association between sex and the presence of long COVID-19 [32,42,43,44,45,46,50].

Seven articles (n = 386,234 COVID-19 patients who recovered from acute SARS-CoV-2 infection) were included in the meta-analysis due to their similarities in study design, definition of long COVID-19, as well as similarities in data presentation [32,42,43,44,45,46,50]. The meta-analysis revealed that female sex was significantly associated with nearly 50% higher risk (OR 1.48, 95% CI 1.17 to 1.86, Q = 17.2, *p* = 0.01, I2: 65%, Figure 4) of long COVID-19 symptomatology.

### 3.4. Medical Comorbidities and Post-COVID-19 Condition 

A total of 12 articles with 677,045 COVID-19 survivors were analyzed for association between long COVID-19 and comorbidities (Table 4) [29,39,44,52,56,57,58,59,60,61,62,63]. Four comorbidities were included: pulmonary disease (n = 4), diabetes (n = 1), obesity (n = 6), and organ transplantation (n = 1). Data were presented as means, medians, percentages, odds ratio (OR), and incident rate ratio (IRR). One study used mean [61], one used both median and percentage [59], three used percentage only [44], five used OR [29,39,52,55,60], and two used both OR and IRR [57,59].

Three articles on pulmonary disease revealed an association between asthma and longer symptom duration among patients recovering from COVID-19 [29,44,60]. However, both asthma and chronic pulmonary disease were not associated with long COVID-19 in one study [52]. For diabetes, no difference was found in the number of long COVID-19 symptoms among diabetic and non-diabetic patients [57]. For obesity, all six articles noted that this metabolic disease was associated with worse health due to increased number of long COVID-19 symptoms [39,59], longer persistence of symptoms [56,63], more presence of pathological pulmonary limitations [61], and metabolic abnormalities [58]. Meanwhile, one study on kidney transplant patients revealed that patients have higher susceptibility to developing long COVID-19 symptoms, although this did not affect mortality rate [62].

### 3.5. Risk of Bias

**Figure 5:**
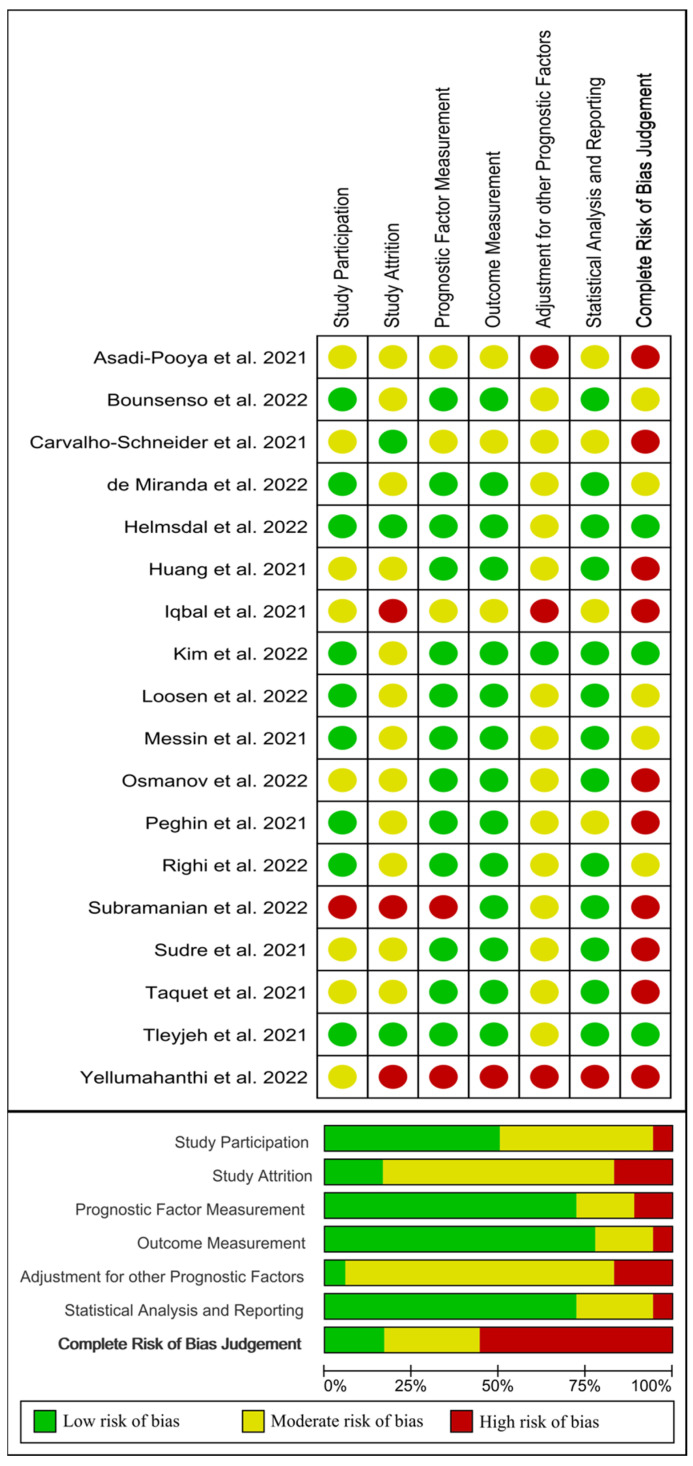
Plot of the risk of bias of those studies investigating age as a risk factor of long COVID-19 [10,26,27,28,29,30,31,32,33,35,36,37,38,39,40,41,42,43].

From out of 18 papers evaluating age as risk factor [26,27,28,29,30,31,32,33,34,35,36,37,38,39,40,41,42,43], three [35,41,43] were classified as low risk of bias (green), five [26,37,38,39,40] as moderate risk of bias (yellow), and the remaining ten [27,28,29,31,32,33,34,36,42,51] as high risk of bias (red). Figure 5 visually graphs that the most frequent risk of bias was adjustment for other prognostic factor (i.e., if important potential confounding factors were appropriately accounted for), which was properly performed in just one study [41].

**Figure 6:**
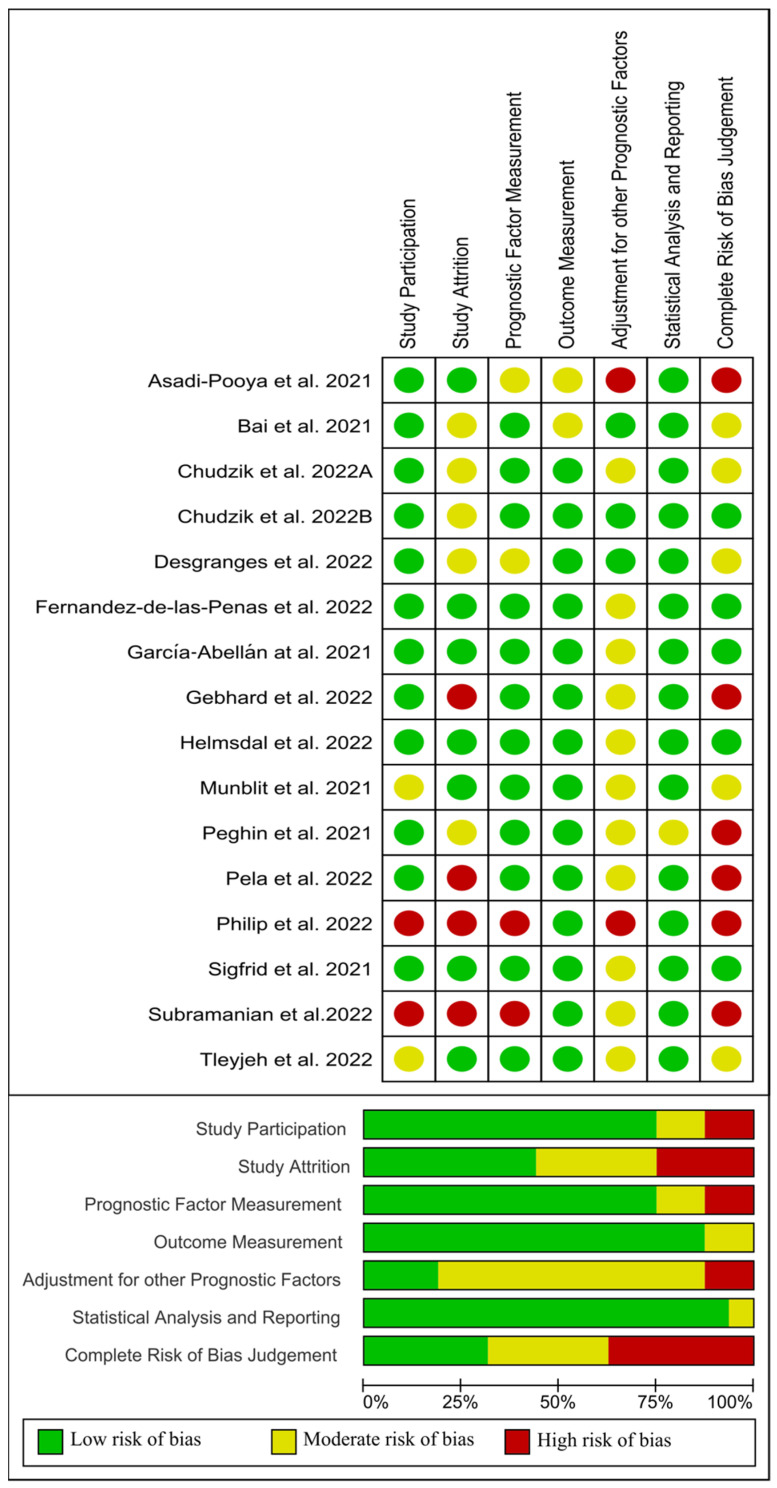
Plot of the risk of bias of those studies investigating sex as a risk factor of long COVID-19 [1,32,42,43,44,45,46,47,48,49,50,51,52,54,55,56].

On the other hand, from 16 papers evaluating sex as a risk factor [32,42,43,44,45,46,47,48,49,50,51,52,53,54,55,56], four studies [45,50,53,55] were classified as low risk of bias (green), five [46,49,52,54,56] as moderate risk of bias (yellow), and the remaining seven [32,42,43,44,47,48,51] as high risk of bias (red). Figure 6 visually graphs that the most frequent risk of bias in this group of studies were adjustment for other prognostic factors and study attrition (i.e., the representativeness of the participants with follow-up data with respect to those originally enrolled in the study, selection bias).

**Figure 7:**
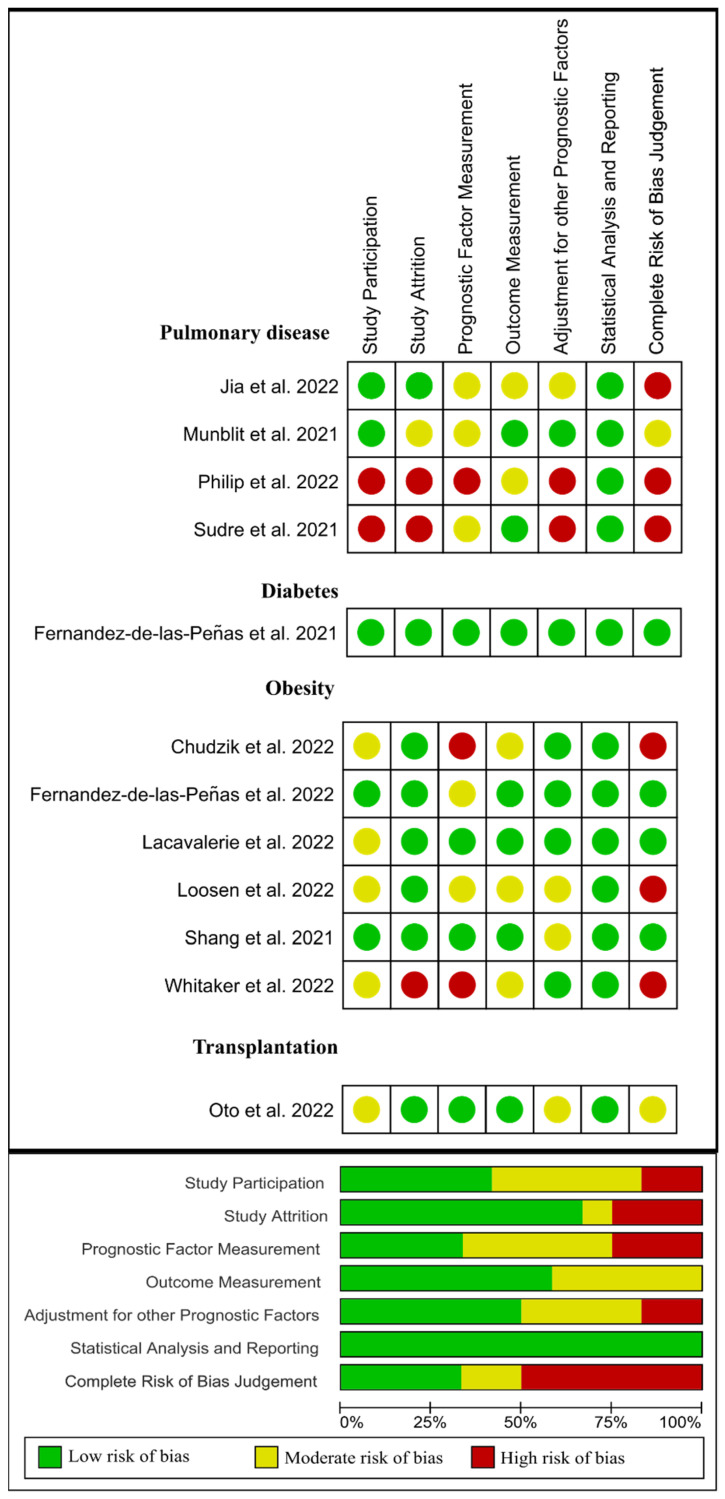
Plot of the risk of bias of those studies investigating medical comorbidities as a risk factor of long COVID-19 [4,29,39,44,52,53,56,58,60,61,62,63].

Lastly, from 12 papers evaluating previous medical comorbidities as a risk factor [29,39,44,52,56,57,58,59,60,61,62,63], four [57,58,59,61] were classified as low risk of bias (green), two [52,62] as moderate risk of bias (yellow), and the remaining six [29,39,44,56,60,63] as high risk of bias (red). Figure 7 visually graphs that the most frequent risk of bias in this group of studies was concerned prognostic factor measurement (i.e., if the prognostic factors were measured in a similar way for all the participants).

## 4. Discussion

This systematic review and meta-analysis explored the association of long COVID-19 with risk factors not directly related to an acute SARS-CoV-2 infection (i.e., pre-infection factors), including age, sex, or previous comorbidities. The results support that female sex may be a predictor of long COVID-19 while old age was reported to be associated with long COVID-19 in single studies; however, the pooled evidence was not significant. Finally, prior medical comorbidities can also be potential predictors of long COVID-19 symptoms. These results should be considered with caution because most studies exhibited moderate to high risk of bias.

### 4.1. Old Age and Long COVID-19

Old age is an important risk factor of poor outcomes in COVID-19 hospitalization [64]; however, the impact of age on long COVID-19 is controversial. Old age is associated with higher risk of long COVID-19 symptomatology in single studies and in two previous reviews [10,12], but not in the meta-analysis by Maglietta et al. [11]. Results from our qualitative analysis suggest that older adults can develop more long COVID-19 symptoms than younger adults; however, this assumption was not supported when pooling data into a meta-analysis. We conducted two meta-analyses, the first one categorizing those adults older than 60 years (Figure 2), and a second one considering age as a continuous variable (Figure 3); neither analysis revealed an association between old age and risk of developing long COVID-19. Nevertheless, the number of studies pooled in our analyses of age was notably limited (n = 3). Our data are consistent with the meta-analysis of Maglietta et al. [11] but disagree with Thompson et al. [12]. Several differences can explain the discrepancy with Thompson et al. [12]. It is possible that the use of a different definition of long COVID-19 by these authors [12] can lead to inconclusive comparisons of results. In addition, Thompson et al. [12] did not pool data of age and long COVID-19 into a meta-analysis, but only calculated regression of proportions of subjects at each age group developing long COVID-19 symptoms. The significance of old age as a risk factor for long COVID-19 development requires further investigation. In fact, just three out of eighteen papers (16%) analyzing age as prognostic factor showed low risk of bias. The most significant bias of these studies was the proper control of other cofounding factors observed in older people, i.e., higher presence of medical comorbidities, or longer hospitalization stay, which can also be associated with long COVID-19.

### 4.2. Female Sex and Long COVID-19

Sex is another important risk factor which has been studied in relation to COVID-19 and long COVID-19. Evidence supports that men and women exhibit the same probability of being infected by SARS-CoV-2; however, males are at a higher risk of worse outcomes and death than females during the acute phase of infection [65]. Results from our systematic review and meta-analysis support that female sex may be associated with higher risk of developing long COVID-19 (OR 1.48, 95% CI 1.17 to 1.86). Our results are similar to those previously observed by Maglietta et al. [11], who also reported that female sex was associated with long COVID-19 symptoms (OR1.52, 95% CI 1.27–1.82), and with results (OR1.60, 95% CI 1.23–2.07) previously reported by Thompson et al. [12]. Based on available data, females are more vulnerable to develop long COVID-19 than males. Hence, considering sex differences in diagnosis, prevention and treatment are necessary, and fundamental steps towards precision medicine in COVID-19 [66]. Biological (i.e., hormones and immune responses), and sociocultural (i.e., sanitary-related behaviors, psychological stress, and inactivity) aspects play a significant role in creating sex-differences in long COVID-19 symptoms [48], although mechanisms behind increased risk of long COVID-19 in females remain unknown and warrant investigation. 

### 4.3. Medical Comorbidities and Long COVID-19

Such as with old age, the presence of prior medical comorbidities (e.g., hypertension, obesity, diabetes, chronic kidney disease, cerebrovascular disease, chronic obstructive pulmonary disease, or cardiovascular disease) is known to induce a more severe COVID-19 disease progression [67,68]. A potential reason is that such comorbidities can contribute to degradation of angiotensin-converting enzyme 2 (ACE2). Since the SARS-CoV-2 virus uses this receptor as entry pathway in host cells, higher degradation of ACE2 could lead to a long-lasting inflammatory cytokine storm, oxidative stress, and hemostasis activation, which are all hallmarks of severe/critical COVID-19 illness [69]. Nevertheless, this hypothesis is not yet supported by the literature. 

The current qualitative analysis suggests that prior comorbidities may contribute the risk of developing long COVID-19. Among different comorbidities, obesity seems to be associated; however, this assumption should be considered with caution at this stage, since potential cofounding factors, particularly those related to hospitalization (obese patients have more severe COVID-19 disease and higher hospitalization rates than non-obese patients), were not properly controlled in these studies. Moreover, the association of long COVID-19 with other medical comorbidities such as diabetes or transplants was only investigated in one prior study. 

### 4.4. Strengths and Limitations

The results of this systematic review and meta-analysis should be considered according to potential strengths and limitations. Among the strengths, we conducted a systematic search of all the currently available evidence on factor not related to an acute SARS-CoV-2 infection but associated with higher risk of developing long COVID-19. This led to identification of thirty-eight studies. Second, this is the first time that several medical comorbidities have been systematically investigated as risk factors of long COVID-19. 

One of the limitations is the lack of a consistent definition of long COVID-19 in available literatures. We included all identified studies within the qualitative analysis, but only those using the definition by Soriano et al. [2] of long COVID-19 were included in the meta-analyses. This assumption led to a small number of studies in the meta-analyses. Future studies using a more consistent definition of long COVID-19 are needed for improved quantification of the results. Another limitation is the lack of differentiation of risk factors between hospitalized and non-hospitalized patients. Similarly, no study investigating risk factors considered the SARS-CoV-2 variants of concern. Therefore, studies identifying long COVID-19 risk factors not directly associated with SARS-CoV-2 infection differentiating between hospitalized and non-hospitalized patients, and among different SARS-CoV-2 variants of concern are now needed. Finally, it should be considered that this systematic review and meta-analysis only investigated risk factors not associated with an acute SARS-CoV-2 infection. Other potential SARS-CoV-2-associated factors, such as severity of disease during the acute phase of infection or the number of COVID-19-associated onset symptoms have also been preliminarily identified as risk factors associated with long COVID-19 symptoms, particularly with respiratory symptoms [11]. Similarly, it is possible that some long COVID-19 symptoms can also be related to hospitalization factors which were also not investigated in this review.

## 5. Conclusions

The current review demonstrates that female sex and previous medical comorbidities may be predisposing factors for the development of long COVID-19 symptomatology. The current literature does not conclusively confirm that old age would significantly influence long COVID-19 risk. These results should be considered with caution due to moderate to high risk of bias in most published studies. These findings highlight the need for further research with improved control of confounding factors and use of a consistent and validated definition of long COVID-19.

## Figures and Tables

**Table 1:** Database formulas during literature search.

PubMed Search Formula
#1 “post-acute COVID-19 syndrome” [MeSH Terms] OR “long COVID-19” [All Fields] OR “long COVID-19 symptoms” [All Fields] OR “long hauler” [All Fields] OR “post-COVID-19” [All Fields] OR “post-acute COVID-19 symptoms” [All Fields] OR “COVID-19 sequelae” [All Fields] #2 “age” [All Fields] #3 “sex” [MeSH Terms] OR “sex” [All Fields] #4 “comorbidity” [MeSH Terms] OR (“transplants” [MeSH Terms] OR “transplantation” [MeSH Terms] OR transplant [All Fields]) OR (“obesity” [MeSH Terms] OR obesity [All Fields]) OR (“diabetes mellitus” [MeSH Terms] OR “diabetes insipidus” [MeSH Terms] OR diabetes [All Fields]) OR (“hypertension” [MeSH Terms] OR hypertension [All Fields]) OR (“lung diseases” [MeSH Terms] OR pulmonary disease [All Fields]) OR (“asthma” [MeSH Terms] OR asthma [All Fields]) OR (“pulmonary disease, chronic obstructive” [MeSH Terms] OR COPD [All Fields]) #5 #1 AND #2 #6 #1 AND #3 #7 #1 AND #4
MEDLINE/CINAHL (via EBSCO) Search Formula
#1 “post-acute COVID-19 syndrome” OR “long COVID-19” OR “long COVID-19 symptoms” OR “long hauler” OR “post-COVID-19” OR “post-acute COVID-19 symptoms” OR “COVID-19 sequelae” #2 “age” #3 “sex” #4 “comorbidity” OR “transplants” OR “transplantation” OR “obesity” OR “diabetes mellitus” OR “diabetes” OR “hypertension” OR “pulmonary disease” OR “asthma” OR “chronic obstructive pulmonary disease” #5 #1 AND #2 #6 #1 AND #3 #7 #1 AND #4
WOS (EMBASE)/Web of Science Search Formula
(“post-acute COVID-19 syndrome” OR “long COVID-19” OR “long COVID-19 symptoms” OR “long hauler” OR “post-COVID-19” OR “post-acute COVID-19 symptoms” OR “COVID-19 sequelae” AND ((“age”) OR (“sex”) OR (“comorbidity” OR “transplants” OR “transplantation” OR “obesity” OR “diabetes mellitus” OR “diabetes” OR “hypertension” OR “pulmonary disease” OR “asthma” OR “chronic obstructive pulmonary disease”))

**Table 2:** Studies investigating the effect of age in long COVID-19 symptomatology [10,26,27,28,29,30,31,32,33,35,36,37,38,39,40,41,42,43].

Author	Country Study Period	Study Design Sample Size	Age	Symptoms Assessment	Post-COVID-19 Symptoms	Main Findings
Buonsenso et al., 2022	Italy 1 April 2020–31 April 2021	Prospective cohort n = 507	Adults, 44 y	ISARIC Global COVID-19 protocol EQ-5D-5L	Headache, Malaise, Fatigue	Probability of being fully recovered: 1–3 months Adults 0.83 (0.38), *p* = 0.001 6–9 months Adults 0.83 (0.38), *p* = 0.016
Yellumahanthi et al., 2022	USA 13 March 2020–12 March 2021	Prospective cohort n = 53	18–64 y (n = 38) ≥65 y (n = 15)	Self-reported questionnaire three months after	Fatigue, Brain fog Shortness of breath, Joint pain, Loss taste/smell, Anxiety/Depression, Hair loss, Sleep disturbances, Cough	18–64 y Symptoms present n = 20 Symptoms absent n = 18 >65 y Symptoms present n = 7 Symptoms absent n = 8 *p* = 0.696
Huang et al., 2021	China 16 June 2020–13 September 2020	Cohort study n = 1733	mean 57 y	EQ-5D-5L	Fatigue, Sleep difficulties, Anxiety/Depression	Per 10-year increase im age—Risk of Fatigue OR 1.17, 95% CI 1.07–1.27 *
Sudre et al., 2021	UK, USA, Sweden March 2020–December 2020	Prospective cohort n = 8364	Positive for SARS-CoV-2 42 y (IQR 32–53) Negative for SARS-CoV-2 42 y (IQR 32–53)	COVID-19 Symptoms Study app	Abdominal pain, Chest pain, Sore throat, Shortness of breath, Fatigue, Hoarse voice, Diarrhea, skipped meals, Cough, Muscle pain, Loss of smell, Headache	OR (95% CI)—18–30 y 30–40 y—OR from 2.11 to 4.12 * 40–50 y—OR from 2.24 to 4.35 * 50–60 y—OR from 6.65 to 11.49 * 60–70 y—OR from 6.53 to 14.0 * ≥70 y—OR from 5.46 to 18.56 *
Asadi-Pooya et al., 2021 #	Iran February 2020 –February 2021	Cross-Sectional n = 58	Mean age 12.3 y (SD 3.3)	Telephone interview	Fatigue, Shortness of breath, Exercise intolerance, Walking intolerance, Cough, Sputum, Sleep difficulty, Muscle/Joint pain, Headache, Chest pain, Palpitation, Loss of smell, Sore throat, Dizziness	OR 1.314 (95% CI 1.043–1.656), *p* = 0.002 *
Taquet et al., 2021	USA January 2020–December 2020	Retrospective cohort n = 388,067	COVID-19 (unmatched) Mean age 46.3 y (SD 9.8) COVID-19 (matched) Mean age 39.4 y (SD 18.4) Influenza (matched) Mean age 38.3 y (SD 19.7)	Electronic health records	Breathlessness, Fatigue/malaise, Chest pain, Throat pain, Headache, Abdominal pain, Myalgia, Cognitive symptoms, Anxiety/depression	6-month incidence of long COVID-19 symptoms % (95% CI) 10–21 y—55.06 (54.34–55.77) 45–64 y—58.92 (58.24–59.59) ≥65 y—61.05 (60.29–61.81)
Peghin et al., 2021	Italy March 2020–November 2020	Cohort n = 599	Mean age 53 y (SD 15.8)	Questionnaire via telephone interview	Dyspnea, Cough, Fatigue, Chest pain, Anosmia/Dysgeusia, Headache, Sleep disorders, Neurological Disorders, Brain Fog, Anxiety/Depression, Skin lesion, Gastrointestinal Dis., Hair loss, Ocular involvement	41–60 vs.18–40 y OR 1.0 (95% CI 0.6–11.6), *p* = 0.9 >60 vs. 18–40 y OR 1.03 (95% CI 0.6–1.7), *p* = 0.9 >60 vs. 41–60 y OR 1.04 (95% CI 0.67–1.6), *p* = 0.8
Carvalho-Schneider et al., 2021	France March 2020–August 2020	Cohort n = 150	Mean age 49 y (IQR 34–64)	Telephone interviews	Dyspnea Chest pain Palpitations Anosmia/Ageusia Headache Cutaneous signs Arthralgia/Myalgia Digestive disorders Fever Sick leave	One or more long COVID-19 symptom n (%) D30 (n = 150) * <30 y—7 (6.8) 30–39 y—21 (20.4) 40–49 y—24 (23.3) 50–59 y—28 (27.2) 60–69 y—11 (10.7) *p* = 0.06 D60 (n = 130) * <30 y—4 (4.7) 30–39 y—19 (22.1) 40–49 y—23 (26.7) 50–59 y—21 (24.4) 60–69 y—10 (11.6) *p* = 0.026
Iqbal et al., 2021	Pakistan September 2020–December 2020	Cross Sectional n = 158	Mean age 40.1 y (SD 12.42)	Questionnaire	Fatigue, Sleep quality, Anxiety/Depression Dyspnea, Joint pain, Loss of smell/taste, Cough, Loss Hair, Headache, Chest pain, Brain fog, Blurred vision, Tinnitus	Relation of age with post-COVID-19 Dyspnea (*p* = 0.007) * Cough (*p* < 0.001) * Joint pain (*p* < 0.001) * Chest pain (*p* < 0.001) *
Tleyjeh et al., 2021	Saudi Arabia May 2020–January 2021	Prospective Cohort n = 222	Mean age 52.5 y (IQR 38.52–66.42)	Structured interview via phone call	Insomnia, Fever, Fatigue, Joint pain, Muscle pain, Memory loss, Headaches, Loss of taste, Abdominal pain, Nausea/Vomiting, Diarrhea, Loss of smell, Sore throat, Runny nose, Chest pain, Cough, Shortness of breath	Hazard model of new or persistent symptoms at follow-up (n = 222) Adjusted HR (95% CI) 0.99 (0.98–1.01), *p* = 0.38
Osmanov et al., 2022 #	Russia April 2020–February 2021	Prospective Cohort n = 518	Mean age 10.4 y (IQR 3–15.2)	Telephone Interview— 1.0 ISARIC COVID-19 Health and Wellbeing Follow-Up Survey for Children	Respiratory symptoms, Neurological symptoms, Sleep problems, Gastrointestinal Dermatological Cardiovascular Fatigue Musculoskeletal	Presence of any persistent symptom at time of follow-up (n = 127) 2–5 y—OR 0.93 (95% CI 0.38–2.22) 6–11 y—OR 2.57 (95% CI 1.29–5.36) * 12–18 y—OR 2.52 (95% CI 1.34–5.01) *
Righi et al., 2022	Italy February 2020 –February 2021	Prospective Cohort n = 465	Mean age 56 y (IQR 45–66)	Questionnaire	Cough, Diarrhea, Fatigue, Myalgia, Anosmia, Dysgeusia, Breathlessness	Persistence of symptoms at 9-month follow-up >50 y—OR 2.5 (95% CI 1.28–4.88), *p* = 0.007 * Persistence of fatigue at 9-month follow-up >50 y—HR 0.98 (95% CI 0.97–0.99)
de Miranda et al., 2022	Brazil March 2020–November 2021	Longitudinal study n = 646	Mean age 50.3 y (SD 15.8)	In person or virtual interview	Sore throat, Runny nose, Sputum, Skin lesion, Tachycardia, Vertigo, Chest pain, Joint pain, Diarrhea, Anxiety, Insomnia, Myalgia, Headache, Loss of smell/taste, Dyspnea, Fatigue	Mild COVID-19: 59.3% of 329 patients developed symptoms—<60 y: n = 162 (83.1%) Severe COVID-19: 33.1% of 260 patients developed symptoms ≤60 y old: n = 48 (55.8%) >60 y old: n = 38 (44.2%)
Loosen et al., 2022	Germany 1 March 2020–31 March 2021	Cross-sectional n = 50,402	Mean age 48.8 y (SD 19.3)	Medical record data from the Disease Analyzer database	Fatigue, Abnormalities of breathing, Disturbances of smell/taste, Disturbances in attention	≤30 years/COVID-19 patients: n = 10,443 Patients developing long COVID-19: n = 213 31–45 years/COVID-19 patients: n = 12,963 Patients developing long COVID-19: n = 379 46–60 years/COVID-19 patients: n = 14,424 Patients developing long COVID-19: n = 664 >60 years/COVID-19 patients: n = 12,572 Patients developing long COVID-19: n = 452
Messin et al., 2021	France March 2020–October 2020	Retrospective observational n = 74 With persistent symptoms: n = 53 Without persistent symptoms: n = 21	Mean age: 54.7 y (SD 16.9)	Telephone interview	Asthenia, Dyspnea, Anxiety, Anosmia, Ageusia, Nasal obstruction, Rhinorrhea, Sneezing, Odynophagia, Dysphonia, Chest pain, Palpitations, Headache, Dizziness, Drowsiness, Neuropathic pain, Depressive syndrome, Memory impairment, Attention deficit, Hair loss Diarrhea, Cough, Pain, Erectile dysfunction	18–30 years—number (%) Symptoms: 5 (9.4)/No symptoms: 4 (19.1) 31–40 years—number (%) Symptoms: 8 (15.1)/No symptoms: 7 (33.3) 41–50 years—number (%) Symptoms: 8 (15.1)/No symptoms: 6 (28.6) 51–60 years—number (%) Symptoms: 9 (17)/No symptoms 0 61–70 years—number (%) Symptoms: 14 (26.4)/No symptoms: 0 >71 years—number (%) Symptoms: 9 (17)/No symptoms: 4 (19.1)
Kim et al., 2022	Korea 31 August 2020–2 March 2021	Prospective cohort n = 170 With persistent symptoms: n = 129 Without persistent symptoms: n = 41	Median age: 51 y (IQR 37–61)	Individualized questionnaire	Fever, Myalgia, Cough, Arthralgia, Fatigue, Sore throat, Rhinorrhea, Chest pain, Dyspnea, Palpitation, Arrhythmia, Headache, Cognitive dysfunction, Dizziness, Insomnia, Depression/Anxiety, Vomiting, Diarrhea Anosmia, Ageusia, Tinnitus, Alopecia, Skin rash, Paresthesia	20–29 years—number (%) Symptoms: 19 (14.7)/No symptoms: 10 (24.4) 30–39 years—number (%) Symptoms: 18 (14)/No symptoms: 6 (14.6) 40–49 years—number (%) Symptoms: 17 (13.2)/No symptoms: 9 (22) 50–59 years—number (%) Symptoms: 35 (27.1)/No symptoms: 9 (22) 60–70 years—number (%) Symptoms: 40 (31)/No symptoms: 7 (17.1)
Subramanian et al., 2022	United Kingdom 31 January 2020–15 April 2021	Retrospective matched cohort study Non-hospitalized COVID-19 survivors n = 486,149 Matched patients with no evidence of COVID-19 n = 1,944,580	Patients infected with SARS-CoV-2 Mean age 44.1 y (SD 17.0) Comparator cohort Mean age 43.8 y (SD 16.9)	Interviews and questionnaires	A total of 62 symptoms were significantly associated with SARS-CoV-2 infection after 12 weeks: Anosmia, Hair loss, Sneezing, Ejaculation difficulty, Reduced libido, Shortness of breath at rest, Fatigue, Chest pain, Hoarse voice, Fever	18–29 years (n = 95,969) With symptoms: n (%) 6932 (7.2) 30–39 years (n = 78,302) With symptoms: n (%) 5805 (7.4) 40–49 years (n = 75,349) With symptoms: n (%) 5784 (7.7) 50–59 years (n = 73,262) With symptoms: n (%) 5485 (7.5) 60–69 years (n = 35,932) With symptoms: n (%) 2790 (7.8) ≥70 years (n = 25,323) With symptoms: n (%) 3073 (12.1)
Helmsdal et al., 2022	Faroe Islands March 2020–January 2022	Cohort n = 180	Mean age 40 y (SD 19.4)	Standardized questionnaire via telephone interview	Fatigue, loss taste, loss smell, Headache, Skin rashes, Arthralgia, Dyspnea, Myalgia, Rhinorrhea, Chest tightness, Cough, Diarrhea, Nausea, Anorexia, Chills, Fever, Sore throat	Prevalence (%) of long COVID-19 (n = 170) at 23-months Mean (SD) Age at symptom onset * Symptoms (n = 65) age: 45.1 (18.5) No symptoms (n = 105) age: 36.9 (19.3) *p* = 0.03 Persistent symptoms vs. No symptoms—n (%) 0–17 y—4 (6.2) vs. 17 (16.2) 18–34 y—16 (24.6) vs. 34 (32.4) 35–49 y—17 (26.2) vs. 22 (21.0) 50–67 y—18 (27.7) vs. 25 (23.8) >67 y—10 (15.4) vs. 7 (6.7) *p* = 0.1

* Statistically significant (*p* < 0.05); # Data from children were not considered in the analyses. y: years; SD: standard deviation.

**Table 3:** Studies investigating the effect of sex in long COVID-19 symptomatology [32,42,43,44,45,46,47,48,49,50,51,52,53,54,55].

Author	Country Study Period	Study Design Sample Size	Age	Symptoms Assessment	Post-COVID-19 Symptoms	Main Findings
Bai et al., 2021	Italy 15 April 2020–15 December 2020	Prospective Cohort n = 377 Female 137	Median age 57 y (IQR 49–68)	Interview and physical examination Impact of Event Scale-Revised (IES-R)	Anosmia, Dysgeusia, Gastrointestinal symptoms, Fever, Joint pain, Myalgia, Dyspnea at rest, Exertional dyspnea, Fatigue, Brain fog, PTSD, Depression, Anxiety	Female Sex Risk Long COVID-19 OR 2.78 (95% CI 1.68–4.62) * Long COVID-19 AOR3.32 (95% CI 1.78–6.17) *
Pela et al., 2022	Italy Follow-up: May 2020–March 2021	Cohort n = 223 Female 89	Mean age 59 y (SD 13)	Retrospective Medical records Prospective Long COVID-19-19 reevaluation	Dyspnea, Cough, Fatigue, Chest pain, Palpitations, Myalgia, Sleep disturbance	Female Sex Risk Dyspnea OR 2.35 (95% CI 1.12–4.94) * Fatigue OR6.72 (95% CI 2.34–19.26) * Chest pain OR 2.04 (95% CI 1.00–4.15) * Palpitation OR 2.30 (95% CI 1.14–4.65) *
Sigfrid et al., 2021	UK NR	Prospective Cohort n = 327 Female 135	Media age 60 y (IQR 51.7–67.7)	Washington group short scale MRC Dyspnea Scale EQ5D-5L	Fatigue, Breathlessness, Sleep problems, Headache, Limb weakness, Muscle pain, Joint pain, Dizziness, Palpitations, Ocular problems, Stomach pain, Diarrhea, Cough, Chest pain, Loss of smell, Fever, Loss of taste, Nausea, Vomiting, Skin rashes	Female Sex < 50 years Risk Long COVID-19 (AOR 5.09, 95% CI 1.64–15.74) * Fatigue (AOR 2.06, 95% CI 0.81–3.31) Breathlessness (AOR 7.15, 95% CI 2.24–22.83) *
Fernandez-de-las-Peñas et al., 2022	Spain 10 March 2020–31 May 2020	Cross-sectional n = 1969 Female 915	Mean age 61 y (SD 16)	Telephone interview	Fatigue, Dyspnea at rest, Dyspnea at exertion, Pain, Memory loss, Brain fog, Concentration loss, Hair loss, Palpitations, Skin rashes, Diarrhea, Voice problems, Gastrointestinal problems, Ageusia, Anosmia, Ocular Problems, Throat pain, Anxiety/Depression, Sleep quality	Female Sex Risk Symptoms (AOR 2.54, 95% CI 1.67–3.86) * Fatigue (AOR 1.51, 95% CI 1.04–2.20) * Dyspnea rest (AOR 1.42, 95% CI 1.08–1.88) * Dyspnea exertion (AOR 1.4, 95% CI 1.10–1.79) * Pain (AOR 1.34, 95% CI 1.05–1.72) * Hair loss (AOR 4.52, 95% CI 2.78–7.36) * Ocular problems (AOR 1.98, 95% CI 1.18–3.31) * Depression (AOR 1.60, 95% CI 1.00–2.57) * Sleep quality (AOR 1.63, 95% CI 1.09–2.43) *
Gebhard et al., 2022	Switzerland February 2020–December 2020	Prospective cohort n = 2927 Female 1346	NR	Self-reported questionnaires	Dyspnea, Reduced exercise performance, Changes in smell and taste	Females reported at least one persistent symptom than males (43.5% vs. 32.0%, *p* < 0.001) The higher prevalence of PASC in females was observed in both outpatients (40.5% in females vs. 25.4% in males, *p* < 0.001) and hospitalized patients (63.1% in females vs. 55.2% in males, *p* < 0.001)
Tleyjeh et al., 2022	Saudi Arabia May 2020–July 2020	Cohort n = 222 Female 51	Range > 18 y	Medical research council (MRC) dyspnea scale Metabolic equivalent of task (MET) score Chronic fatigability syndrome questionnaire	Breathlessness, Exercise intolerance, Chronic fatigue, Poor mental well-being	Female Sex Risk Exertional Dyspnea OR4.36 (95% CI 2.25–8.46) * Lower MET exercise tolerance score OR0.19 (95% CI 0.09–0.42) * Chronic Fatigability Syndrome OR3.97 (95% CI 1.85–8.49) *
Desgranges et al., 2022	Switzerland 26 February–27 April 2020	Prospective cohort n = 418 Female 261	Median age 41 y (IQR 31–54)	Structured and standardized phone survey	Fatigue, Smell or taste disorder, Dyspnea, Headache, Memory impairment, Hair loss, Sleep disorders	Female Sex Risk Symptoms AOR 1.67 (95% CI 1.09–2.56) * Dyspnea AOR1.71 (95% CI 0.93–3.16) Smell/taste disorder AOR 1.9 (95% CI 1.09–3.22) * Fatigue AOR1.61 (95% CI 1.00–2.59) *
García-Abellán at al., 2021	Spain 10 March–30 June 2020	Prospective longitudinal study n = 146 Female 58	Median age 65 y (IQR 55–75)	Self-rated COVID-19 symptom questionnaire (CSQ)	Fatigue, Myalgia, Sweating, Headache, Cough, Difficulty breathing, Congestion, Sore throat, Anosmia, Diarrhea, Vomiting, Abdominal pain	Female Sex Risk Highest COVID-19 symptom questionnaire (CSQ) scores OR 2.41 (95% CI 1.20–4.82) *
Asadi-Pooya et al., 2021	Iran 19 February 2020–20 November 2020	Retrospective observational study n = 4681 Female 2203	Mean age 52 y (SD 15)	Telephone interview	Weakness, Muscle pain, Fatigue, Sleep difficulty, Palpitations, Cough, Brain fog, Walking intolerance	Female Sex Risk Long COVID-19 Symptoms OR1.26 (95% CI1.12–1.43) *
Munblit et al., 2021	Russia 8 April 2020–10 July 2020	Longitudinal cohort study n = 2649 Female 1353	Median age 56 y (IQR 46–66)	Study case report form (CRF) British Medical Research Council (MRC) dyspnoea scale EQ-5D-5L WHODAS 2.0	Fatigue, Breathlessness, Forgetfulness, Muscle weakness, Ocular problems, Hair loss, Sleeping problem	Female Sex Risk Symptoms OR1.83 (95% CI 1.55–2.17) * Fatigue OR1.67 (95% CI 1.39–2.02) * Neurological OR2.03 (95% CI 1.60–2.58) * Mood OR1.83 (95% CI 1.41–2.40) * Dermatological OR3.26 (95% CI 2.36–4.57) * Gastrointestinal OR2.50 (95% CI 1.64–3.89) * Sensory OR1.73 (95% CI 2.06–2.89) * Respiratory OR1.31 (95% CI 1.06–1.62) *
Chudzik et al., 2022	Poland 1 September 2020–30 September 2021	Retrospective cohort n = 2218 Female 1410	Mean age 54 y (SD 13.5)	Health questionnaire	Fatigue, Headache, Cough Brain fog, Dyspnoea, Hair loss, Olfactory dysfunction, Osteoarticular pain	Female Sex Risk Symptoms OR 1.44 (95% CI 1.20–1.72) * Brain fog OR1.15 (95% CI0.88–1.51) Fatigue OR1.06 (95% CI0.89–1.28)
Peghin et al., 2021	Italy March 2020–November 2020	Cohort n = 599 Female 320	Mean age 53 y (SD 15.8)	Questionnaire via telephone interview	Dyspnea, Cough, Fatigue, Myalgia, Chest Pain, Anosmia/Dysgeusia, Headache, Arthralgia, Neurological Disorders Anxiety/Depression, Sleep Disorders, Brain Fog, Skin Lesions, Gastrointestinal Disorders, Hair Loss, Nose Cold, Sneezing, Odynophagia, Ocular Problems	Female Sex Risk Long COVID-19 Symptoms OR 1.55 (95% CI 1.05–2.27) *
Philip et al., 2022	UK October 2020	Retrospective cohort n = 4500	Range age 50–59 y	Asthma UK and British Lung Foundation survey	Fatigue, Breathlessness, Pain (chest or whole body)	No association between female sex and long COVID-19 symptoms
Helmsdal et al., 2022	Faroe Islands March 2020–January 2022	Cohort n= 180 Female 93	Mean age 40 y (SD 19.4)	Standardized questionnaire via telephone interview	Fatigue, affected taste, affected smell, Headache, Arthralgia, Dyspnea, Myalgia, Skin rashes, Rhinorrhea, Chest tightness, Cough, Nausea, Diarrhea, Fever, Sore throat	No association between female sex and long COVID-19 symptoms
Subramanian et al., 2022	United Kingdom 31 January 2020–15 April 2021	Retrospective matched cohort study Non-hospitalized COVID-19 survivors n = 486,149 Matched patients with no evidence of COVID-19 n = 1,944,580	Patients infected with SARS-CoV-2 Mean age 44.1 y (SD 17.0) Comparator cohort Mean age 43.8 y (SD 16.9)	Interviews and questionnaires	Anosmia, Hair loss, Sneezing, Ejaculation difficulty, Reduced libido, Shortness of breath at rest, Fatigue, Chest pain, Hoarse voice, Fever	Female Sex Risk Long COVID-19 Symptoms HR 1.86 (95% CI 1.81–1.90) * aHR 1.52 (95% CI 1.48–1.56) *

WHODAS: Washington disability score and World Health Organization Disability Assessment Schedule. * Statistically significant (*p* < 0.05). NR: not reported; y: years; SD: standard deviation.

**Table 4:** Studies investigating the effect of previous medical comorbidities in long COVID-19 symptomatology [29,39,44,52,53,56,58,59,60,61,62,63].

Author	Country Study Period	Study Design Sample Size	Age	Symptoms Assessment	Post-COVID-19 Symptoms	Main Findings
Diabetes						
Fernandez-de-las-Peñas et al., 2021	Spain 1 March–31 May 2020	Case-control n = 435 Patients n = 145 Control n = 290	Patients Mean age 70.2 y (SD 13.2) Controls) Mean age 70.4 y (SD 13.4)	Hospital medical records Telephonic interview	Fatigue, Dyspnea on exertion and at rest, Pain, Memory loss, Skin rashes, Gastrointestinal dis., Brain fog, Concentration loss, Ageusia, Ocular disorder, Anosmia, Tachycardia, Cough, Headache, Sleep, Depression/Anxiety	Number of post-COVID-19 symptoms (IRR 1.06, 95% CI 0.92–1.24) Fatigue (OR 1.45, 95% CI 0.93–2.25) Dyspnea (OR 0.97, 95% CI 0.64–1.47) Pain (OR 0.951, 95% CI 0.76–1.18) Anxiety (OR 1.30, 95% CI 0.77–2.20) Depression (OR 1.31, 95% CI 0.79–2.17) Poor sleep (OR 1.34, 95% CI 0.89–2.03)
Obesity						
Lacavalerie et al., 2022	France October 2020–June 2021	Retrospective observational n = 80 Patients n = 33 Controls n = 18 n = 29	Patients Mean age 60 y (SD 11) Controls Mean age 50 y (SD 13)	Clinical evaluation with spirometry, cardiopulmonary and exercise testing	Fatigue, Dyspnea, Chest pain, Pulmonary function test, Cardiopulmonary exercise testing	Non-obese vs. obese, *p* value Pulmonary function test Predicted FEV1 (%) 87 ± 13/75 ± 13 *p* = 0.002 * Predicted FVC (%) 82 ± 16/74 ± 14 *p* = 0.04 * TLC (%) 79 ± 9/69 ± 12 *p* = 0.003 * RV (%) 71 ± 25/86 ± 24 *p* = 0.04 * KCO (%)100 ± 11/108 ± 12 *p* = 0.03 * Cardiopulmonary exercise testing Peak VE/VO2 35 ± 5/39 ± 7 *p* = 0.011 * Ventilatory reserve (%) 40 ± 14/25 ± 21 *p* = 0.011 * VE VCO2 slope 34 ± 6/31 ± 4 *p* = 0.045 * Peak SpO2 (%) 98 ± 2/96 ± 3 *p* = 0.036 *
Fernandez-de-las-Peñas et al., 2022	Spain 1 March 2020–31 March 2021	Case-control n = 264 Patients n = 88 Control n = 176	Patients Mean age 52 y (SD 14.5) Controls Mean age 52.2 y (SD 14.2)	Hospital medical records Telephonic interview	Fatigue, Dyspnea, Memory loss, Skin rashes, Brain Fog, Gastrointestinal disorders, Concentration loss, Ageusia, Ocular disorders, Tachycardia, Pain, Anosmia, Headache, Sleep, Depression/Anxiety	Number of post-COVID-19 symptoms (IRR 1.51, 95% CI 1.24–1.84) * Sleep quality (OR 2.27, 95% CI 1.34–3.86) * Fatigue (OR 1.39; 95% CI 0.79–2.43) Dyspnea (OR 1.41; 95% CI 0.79–2.53) Anxiety (OR 1.75, 95% CI 0.82–3.72) Depression (OR 0.83, 95% CI 0.40–1.73)
Loosen et al., 2022	Germany 1 March 2020–31 March 2021	Retrospective Observational n = 50,402	Mean age 48.8 y (SD 19.3)	Medical record	Fatigue, Abnormalities of breathing, Loss of smell and taste, disturbances in attention	Obesity (OR 1.25 95% CI 1.08–1.44) * Hypertension (OR 1.31, 95% CI 1.15–1.48) *
Shang et al., 2021	Wuhan, China 20 February–20 March 2020	Cohort Study n = 118 Patients n = 53 Controls n = 65	Patients Mean age 51 y (IQR 41–58) Controls Mean age 57 y (IQR 48–62)	Interview, Physical exam, Blood sample, Lung function test, CT scan	Shortness of breath, Fatigue, Sleep problems, Joint pain, Smell disorder, Diarrhea, Constipation	No differences in the prevalence of long COVID-19 Symptoms existed between obese and non-obese patients.
Whitaker et al., 2022	UK 15–28 September 2020 27 October–10 November 2020 25 January–8 February 2021 12–25 May 2021	Cohort n = 606,434	Age >18	Online/telephone survey	Tiredness, Tight chest, Sore throat, Sore eyes, Sneezing, Shortness of breath, Fatigue, Runny nose, Skin lesions, Cough, Pain symptoms, Nausea, Vomiting, Loss of taste or smell, Hoarse voice, Headache, Dizziness, Difficulty sleeping, Diarrhoea, Chest pain, Abdominal pain	Persistence of one or more symptoms for 12 weeks or more Obesity (OR1.39, 95% CI 1.32–1.48) *
Chudzik et al., 2022	Poland 1 September 2020–30 September 2021	Retrospective observational n = 2218	Mean age = 53.8 ± 13.5 years	Health questionnaire	Cough, Dyspnea, Fatigue, Hair loss, Olfactory disturbances, Headache, Pain, Brain fog	Presence of overall persistent symptoms Obesity (OR1.16, 95% CI 0.96–1.41) Fatigue (OR 1.49, 95% CI 1.24–1.80) *
Pulmonary Disease						
Sudre et al., 2021	UK 25 March–30 June 2020	Prospective cohort n = 8364 COVID-19 n = 4182 No COVID-19 n = 4182	Mean age 46 y	COVID-19 Symptom Study app1	Abdominal pain, Chest pain, Sore throat, Fatigue, Shortness of breath, Hoarse voice, Delirium, Diarrhea, Fever, Cough, Muscle pain, Anosmia, Headache	Presence of long COVID-19 symptoms Asthma (OR 2.14, 95% CI 1.55–2.96) *
Munblit et al., 2021	Russia 8 April–10 July 2020	Prospective cohort n = 2649	Median age 56 y	ISARIC Long-term Follow-up Study questionnaire	Fatigue, Shortness of breath, Forgetfulness	Asthma and chronic pulmonary disease were not associated with persistent symptoms overall, but asthma was associated with neurological (OR1.95, 95% CI 1.25–2.98) * and chronic pulmonary disease was associated with fatigue (OR 1.68, 95% CI 1.21–2.32) *
Philip et al., 2022	UK October 2020	Retrospective cohort n = 4500 COVID-19 n = 471 No COVID-19 n = 3036 COVID-19 n = 972	Range age 50–59 y	Asthma UK and British Lung Foundation survey	Fatigue, Breathlessness, Pain (chest or whole body)	For many people with asthma, COVID-19 is associated with prolonged symptoms and worsening asthma control
Jia et al., 2022	USA March 2020–February 2021	Prospective cohort n = 637 Patients n = 617 Controls n = 20	Patients Mean age 51 y Controls Mean age 54 y	Survey	Cough, Shortness of breath, Fever, Nausea, Vomiting	Comorbid lung disease, asthma and lower levels of initial IgG response to SARS-CoV-2 nucleocapsid antigen were associated with longer symptom duration (mean days: 55 versus 44 days; *p* = 0.04) *
Transplant						
Oto et al., 2022	Turkey 15 March 2021–11 June 2021	Retrospective cohort n = 944 Patients n = 523 Control n = 421	Mean age 46 y	Survey	Respiratory symptoms	Persistence of respiratory symptoms without increased risk of acute rejection, BK and CMV infection, thromboembolic event or urinary tract infection

* Statistically significant (*p* < 0.05). y: years; SD: standard deviation.

## Data Availability

All data derived from the study are included in the paper.
